# Intubation after rapid sequence induction performed by non-medical personnel during space exploration missions: a simulation pilot study in a Mars analogue environment

**DOI:** 10.1186/s13728-015-0038-5

**Published:** 2015-11-01

**Authors:** Matthieu Komorowski, Sarah Fleming

**Affiliations:** Department of Bioengineering, Imperial College London, London, SW7 2AZ UK; University of Leicester, Maurice Shock Building, University Rd, Leicester, LE1 9HN UK

**Keywords:** Space medicine, Space exploration, Simulation, Anaesthesia, Medical training

## Abstract

**Background:**

The question of the safety of anaesthetic procedures performed by non anaesthetists or even by non physicians has long been debated. We explore here this question in the hypothetical context of an exploration mission to Mars. During future interplanetary space missions, the risk of medical conditions requiring surgery and anaesthetic techniques will be significant. On Earth, anaesthesia is generally performed by well accustomed personnel. During exploration missions, onboard medical expertise might be lacking, or the crew doctor could become ill or injured. Telemedical assistance will not be available. In these conditions and as a last resort, personnel with limited medical training may have to perform lifesaving procedures, which could include anaesthesia and surgery. The objective of this pilot study was to test the ability for unassisted personnel with no medical training to perform oro-tracheal intubation after a rapid sequence induction on a simulated deconditioned astronaut in a Mars analogue environment. The experiment made use of a hybrid simulation model, in which the injured astronaut was represented by a torso manikin, whose vital signs and hemodynamic status were emulated using a patient simulator software. Only assisted by an interactive computer tool (PowerPoint^®^ presentation), five participants with no previous medical training completed a simplified induction of general anaesthesia with intubation.

**Results:**

No major complication occurred during the simulated trials, namely no cardiac arrest, no hypoxia, no cardiovascular collapse and no failure to intubate. The study design was able to reproduce many of the constraints of a space exploration mission.

**Conclusions:**

Unassisted personnel with minimal medical training and familiarization with the equipment may be able to perform advanced medical care in a safe and efficient manner. Further studies integrating this protocol into a complete anaesthetic and surgical scenario will provide valuable input in designing health support systems for space exploration missions.

## Background

The question of the safety of anaesthetic procedures performed by non anaesthetics physicians or even by non medical doctors has long been debated and remains a hot topic [1–6]. We explore here this challenging question in the hypothetical context of an exploration mission to Mars. During these flights, the risk of medical conditions requiring surgical and anaesthetic interventions is significant [7–9]. The exact composition of the medical team for a space exploration mission has not been decided yet, but could be limited to a single crew medical officer, who will not necessarily be a medical doctor [10]. While on Earth, at least in high income countries, anaesthesia techniques are only performed by well accustomed and experienced personnel, during long duration space exploration missions, onboard medical expertise might be lacking, or the crew medical doctor himself could become injured or ill and require anaesthesia [10]. Because of the tremendous distances involved, real time telemedical assistance will not be an available, and the crew will need to rely mainly on itself. In these conditions and as a last resort, personnel with limited medical training may have to perform lifesaving procedures, which could include surgery and therefore anaesthesia under extreme stress, in the most remote setting one can consider.

### Simulating Mars conditions

To date, no anaesthetic technique or human surgery has been performed in space, besides local infiltration [11, 12]. Advanced surgical procedures have however been performed on animals in low Earth orbit [12]. In 2002, a NASA working group concluded that providing safe anaesthesia during and immediately following the flight could be achievable with enough understanding of the physiological changes affecting the patient [13]. In the absence of actual experience in space, ground research and simulation might help outline the most appropriate protocols. The medical world has adopted simulation tools and techniques for the initial and continuous training of medical doctors all over the world, and its benefits on their performance has long been demonstrated [14, 15].

A good simulation setup must match as many features of the target environment as possible [16]. The delivery of anaesthesia during interplanetary space missions will be a highly challenging task, impeded by various factors that fall into three main categories: physiological (negative effects of the space environment on the human body), technical (limited medical equipment) and human (lack of medical expertise) [17]. The Table 1 provides some details regarding these factors and a comparison as to whether they were replicated in our simulation to represent an as near to true space exploration anaesthesia setting. The gravity on Mars being approximately one-third of Earth’s gravity, no specific restrain system is needed for the patient, the equipment or the operator. In weightlessness, restraining would however be required, the surgical field covered with a sterile transparent hood [11].

**Table 1:** Constraints to medical care delivery during a space exploration mission and the simulation setting at MDRS. Adapted from Marshburn and Norfleet [17, 36]

Feature of an actual space exploration mission	Constraint replicated at MDRS?
Physiological changes	
Physiological alterations induced by the space environment: space motion sickness, muscular and bone loss, orthostatic hypotension upon return to gravity, loss of aerobic capacity, immunodeficiency	Partially (cardiovascular deconditioning only)
Poor expected hemodynamic tolerance to hypovolemia, general anaesthesia, mechanical ventilation	Yes
Pharmacodynamics and pharmacokinetics changes	Partially (drugs distribution affected by lower cardiac output)
Reduced wound healing	No
Technical constraints	
Isolation and impossible urgent evacuation	Yes
Communication delay	Yes
Immobilisation of patient, operator and equipment (in weightlessness)	Not applicable
Limited medical equipment and consumables (mass, volume, power requirement)	Yes
Limited choice and volume of IV fluids	Yes
Specific IV fluid infusion system (in weightlessness)	Not applicable
Altered drugs shelf-life	No
Risk of closed environment contamination with gas, liquids or biological substances	No
Lack of blood substitutes	Yes
Management of healthcare waste	Yes
Human factors	
Limited medical skills (especially if crew doctor injured or ill)	Yes
Fading of skills during the flight	No
Psychological stress	Partially

Investigations focusing on anaesthesia delivery in space have been scarce, partly because no existing human model on Earth is able to reproduce the physiological changes occurring in response to weightlessness [18, 19]. A hybrid simulation model was used, in which the astronaut was represented by a torso manikin, whose vital signs and hemodynamic status were emulated using a patient simulator software. This model provided an opportunity to assess airway skills, while emulating the altered physiological state of the microgravity exposed patient, in order to test the hemodynamic and respiratory response to blood loss and general anaesthesia.

**Fig. 1:**
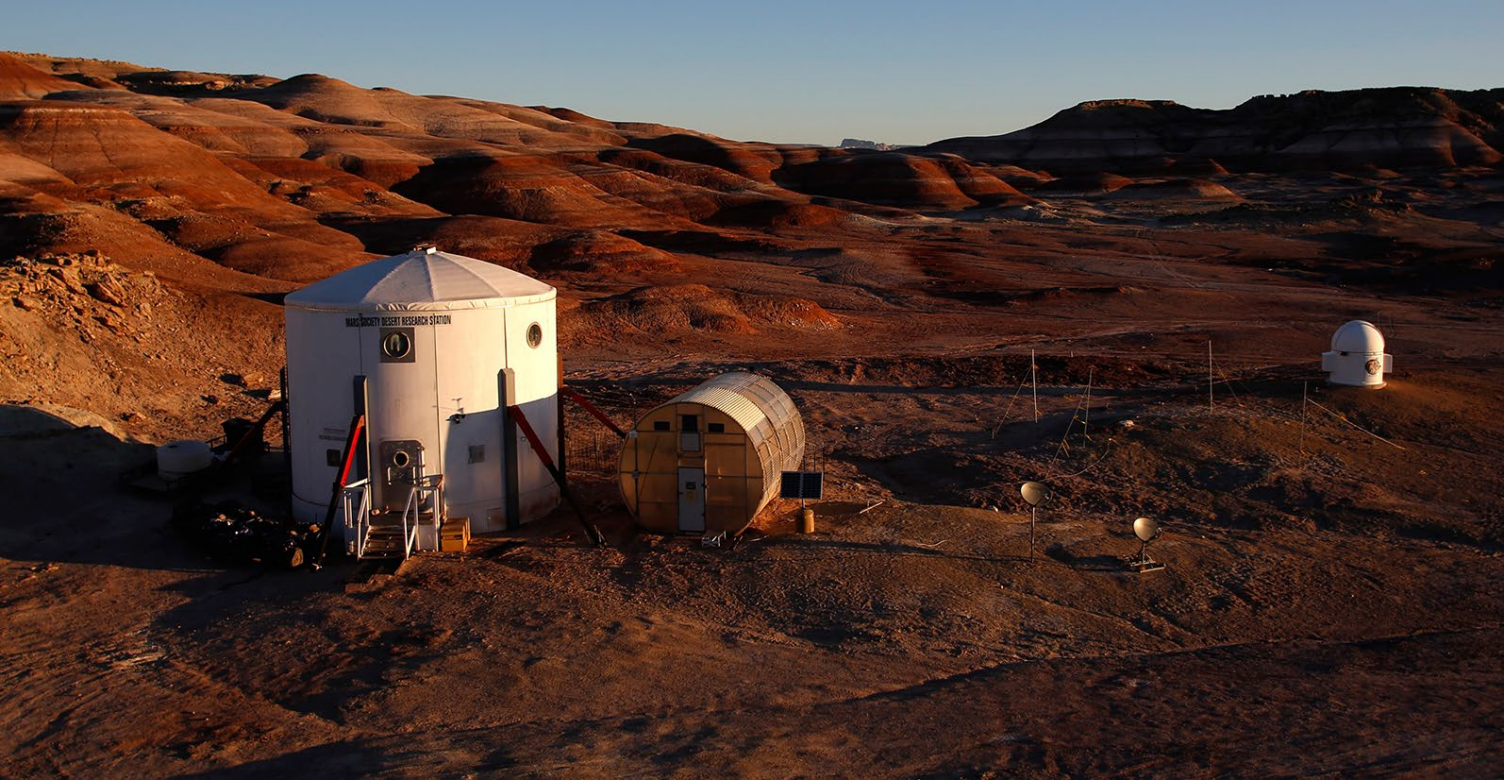
Environment of the Mars Desert Research Station. Credit: James Urquhart

The Mars Desert Research Station (MDRS, Fig. 1), run by the Mars Society, is an isolated facility located in the desert outside of Hanksville, Utah, built for the express purpose of supporting scientific inquiry necessary for the settlement of humans on Mars. Crews of six live at the habitat for 2-week rotations and live as Mars pioneers may 1 day live. No communication with the outside world is possible apart from a very limited access to emails. Crews rely primarily on themselves for any emergency situation, which include medical events.

### Study objectives

The objective of this research was to assess the safety of an intravenous induction and invasive airway management when performed by untrained unassisted personnel on a simulated deconditioned astronaut. The primary outcome was the occurrence of severe complications during the anaesthesia, as detected by the patient simulator software: cardiac arrest, hypoxia (defined by blood saturation <90 %), cardiovascular collapse (mean arterial pressure <65 mmHg), failure to intubate (after 5 min or 5 attempts).

The secondary outcomes consisted of measuring the time to “incision” (time between the beginning of the procedure and the successful intubation) and the apnea time (time between the occurrence of apnea, 30 s after the administration of rocuronium, and the successful intubation).

## Methods

### Subjects

The 6 people of the MDRS crew comprised the commander, the executive officer, the journalist, the engineer, the astronomer and the Health and Safety Officer (HSO, first author, simulation facilitator, consultant level anaesthetist and intensivist by training). Apart from the HSO, no crew member had received previous medical training except for basic life support training for four of them and wilderness first responder training for one person. Informed consent from the five participants and Lens hospital (France) ethical committee approval were obtained.

### Scenario

Acute trauma is considered among the most severe medical conditions that could occur during a space exploration mission and will provide exceptional challenges in terms of medical management [7, 8].

The chosen scenario corresponded to a fall that occurred inside the Mars habitat complicated with an abdominal injury. During maintenance operations on the habitat ventilation system, the expedition medical doctor, a 45 year-old 70 kg male subject fell from a ladder onto a toolbox from which a sharp tool (screwdriver) was sticking out. The screwdriver penetrated his right abdominal flank, causing a severe bleeding. After one liter of blood loss, and precipitated by his impaired cardiovascular status, the patient evolved towards a hemorrhagic shock (blood pressure 57/38 mmHg, heart rate 125 bpm). A lifesaving surgical exploration of the abdomen was urgently needed, and was to be carried out by one of the non-physician crew members.

### Simulation procedure

**Fig. 2:**
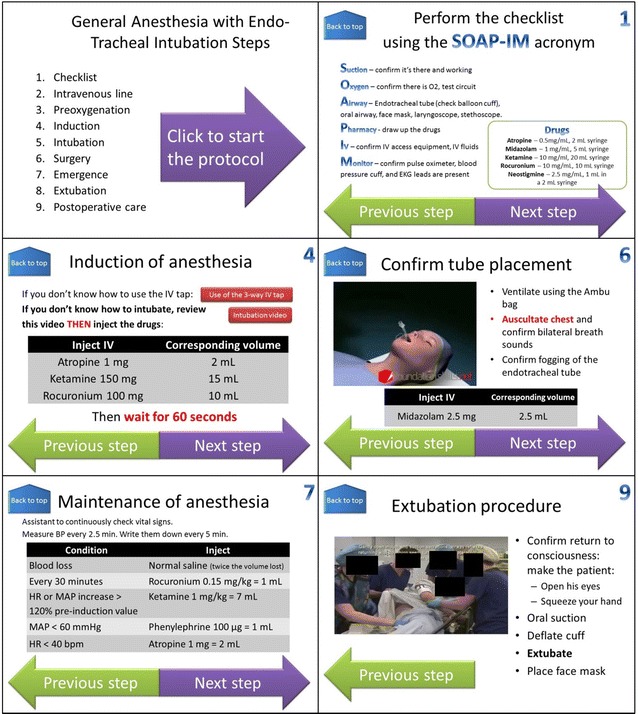
Example of the Powerpoint^®^ slides for the simplified general anaesthesia protocol

In this study, we assessed how crew members with no previous training in anaesthesia performed at the MDRS a simplified rapid sequence induction of general anaesthesia with oro-tracheal intubation on a simulated injured astronaut using a very limited equipment. The patient was represented by a torso mannequin (Ambu^®^ Airway Man), whose vital signs and status were emulated using a patient simulator software (CAE Healthcare^®^ Müse^®^). This particular mannequin allowed testing airway management whilst being lightweight and compact enough to be transported to this remote facility. No physical connection actually linked the mannequin and the software. Instead, the computer tool displayed the simulated vital signs, and the interventions performed by the participant (e.g. IV fluid or drug administration, intubation, initiation of mechanical ventilation…) were manually inputted in real time by the facilitator. The participants did not receive any external assistance during the simulation and could only rely on an interactive computer tool (PowerPoint^®^ presentation, shown in Fig. 2) to guide them through the successive steps of the anaesthesia protocol.

The five participants completed a hands-on familiarization session on the mannequin (duration 20–30 min) and 4–6 days later performed the actual unaided procedure. During the familiarization trials, the subjects were individually introduced with the scenario, the study objectives and the setup including the role of the laptops, the mannequin, the drugs and airway equipment (detailed in Table 2). All participants completed the whole simulation once, following the slides and reviewing the embedded videos (preoxygenation, orotracheal intubation, confirmation of endotracheal tube placement, extubation criteria and procedure) while receiving assistance from the HSO when necessary.

**Table 2:** List of available equipment for the procedure

Equipment category	Items
Monitoring and installation	Operating table (MDRS habitat fold-away table) Monitoring device (simulated by the patient simulator software) Stethoscope
IV access and perfusion	IV line with 3-way tap Two 500 mL crystalloid IV bags
Airway control	Suction tube hand-piece and connection tubing Non-rebreather mask Aitraq^®^ optical laryngoscope, 7.5 mm endotracheal tube with 10 mL syringe to inflate cuff Ventilation bag with Ambu^®^ valve and breathing filter Guedel oral airway
Pretended drugs	Atropine, midazolam, ketamine, rocuronium and sugammadex, pre-drawn in 5 syringes

**Fig. 3:**
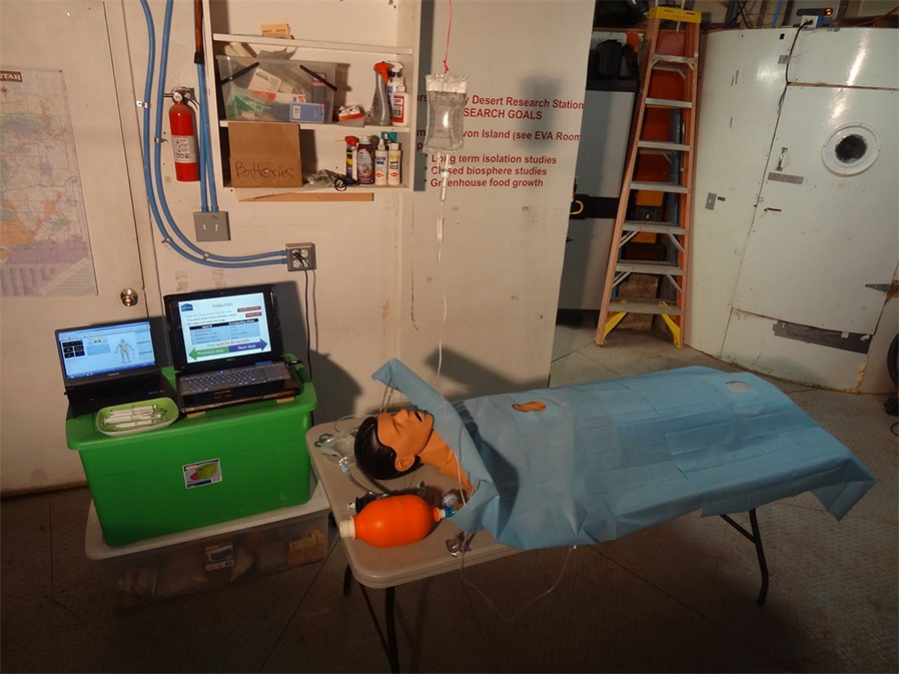
Experimental setting. The laptop on the *left* runs the patient simulator software, the laptop on the *right* depicts the PowerPoint^®^ tool

The experimental setting is depicted in Fig. 3. The simulation began with the mannequin readily installed on the surgical table, a peripheral IV access with a 500 mL bag of normal saline seemingly connected to a vein on his right arm (actually leading to a small container). All the necessary equipment was laid out at proximity and the drugs were drawn up in five labeled syringes.

The steps of the anaesthesia protocol are summarized in Table 3. After performing the checklist (Fig. 2, slide 1), the participants were expected to administer to the patient a volume of IV fluids (normal saline) corresponding to the estimated blood loss (1 l). After restoration of correct haemodynamics, the induction regimen was based on the sequential administration of atropine (1 mg), ketamine (150 mg), rocuronium (100 mg) and midazolam (2.5 mg). This rapid sequence induction protocol is considered the most appropriate for deconditioned astronauts [20]. Endotracheal intubation with an optical larynoscope (Airtraq^®^) was chosen over the conventional Macintosh laryngoscope, because it is associated with higher intubation success rates and shorter intubation times, especially in untrained hands [21, 22]. No ventilator was available at MDRS and the endotracheal tube was connected to an Ambu bag, whilst mechanical ventilation was initiated on the software if requested by the subject. Because the focus of the study was on anaesthetic procedures, the surgery (laparotomy) was not simulated, but basic instructions were given regarding the maintenance phase of the anaesthesia, such as management of blood loss, brady or tachycardia, hypo or hypertension. After a few minutes, the participants were expected to reverse the effect of muscle blockers with sugammadex (1000 mg) and look for return to consciousness, spontaneous ventilation and fulfilment of criteria for extubation. After removal of the endotracheal tube, oxygen administration using a non-rebreather mask was resumed and directives for basic post-operative care were given.

**Table 3:** General anaesthesia protocol steps

Steps
Checklist
Intravenous line (placement not simulated)
Preoxygenation
Optional: video of oro-tracheal intubation
Optional: instruction for use of 3—way IV tap
Induction
Intubation
Surgery (not simulated)
Maintenance phase of anaesthesia
Reversion of muscle blockade
Emergence
Extubation
Postoperative care (not simulated)

The sessions were held privately between the participant and the facilitator and were timed and videotaped. The vital signs were continuously recorded in the simulator software. Technical and non-technical skills of the participants were assessed by the HSO using a predefined scale (Table 4), inspired from the Anaesthetists’ Non-Technical Skills (ANTS) system [23]. The feedback from the participants was also collected using a short questionnaire (Table 5).

**Table 4:** Objective participants’ skill assessment

Skill category	Skill subcategory	Skill assessed
Technical skills	Preparation	1. Performs the checklist, notes missing items
(Max. 20 points)		2. Follows checklist
		3. Identifies hemorrhagic shock
	Procedure	4. Administers the appropriate IV fluid volume
		5. Preoxygenates correctly
		6. Injects the correct sequence of drugs and doses
		7. Intubates correctly
		8. Appropriately confirms tube placement
		9. Reverses the anaesthesia in the recommended manner
		10. Extubates according to instructions
Non-technical skills	Task management	1. Planning and preparing
(Max. 20 points)		2. Prioritizing
	Communication	3. Describes what he/she sees
		4. Describes what he/she does
	Situation awareness	5. Gathering information
		6. Recognizing and understanding
		7. Anticipating
	Decision making	8. Identifies options
		9. Balancing risks and selecting options
		10. Re-evaluating

Each question was given 0 point if the task was not observed, 1 point for tasks partially fulfilled or 2 points for tasks entirely completed. Non-technical skills grading adapted from Anaesthetists’ Non-Technical Skills (ANTS) system [23]

**Table 5:** Feedback questionnaire from the participants. Each question was given −2 points for “bad”, −1 point for “mediocre”, 0 point for “average”, 1 point for “good” and 2 points for “excellent”

Feedback questionnaire to be completed by the participants
1. Quality of the briefing
2. Relevance of the scenario
3. Quality of the simulation equipment
4. Relevance of the research for space exploration
5. Was the training tool understandable?
6. Was the training tool self-sufficient?

### Programming the patient simulator software

High end patient simulator programs, such as the CAE Healthcare^®^ Müse^®^, use complex physiological and pharmacological algorithms able to emulate countless pathological states and realistic responses to a large amount of drugs [24, 25]. On the patient simulator, a 70 kg healthy male adult profile was selected, and his cardiovascular settings were set at levels measured on actual astronauts during and after long-duration spaceflight (Table 6). The use of a patient simulator to reproduce the altered physiological state of the microgravity exposed patient has been tested in the past with encouraging results [26, 27]. Because the effects of a partial gravity environment on the cardiovascular system are not known, we have applied the same settings as in microgravity, in a worst case scenario approach.

**Table 6:** Settings used for the patient profile in the simulator software

Haemodynamic variable	Value
Blood volume	−750 mL (equivalent to −15 %) [18, 28]
Right and left ventricle contractility	−20 % [28]
Baroreflex	−50 % [29, 30]
Systemic vascular resistance	−15 % [31]

## Results

**Fig. 4:**
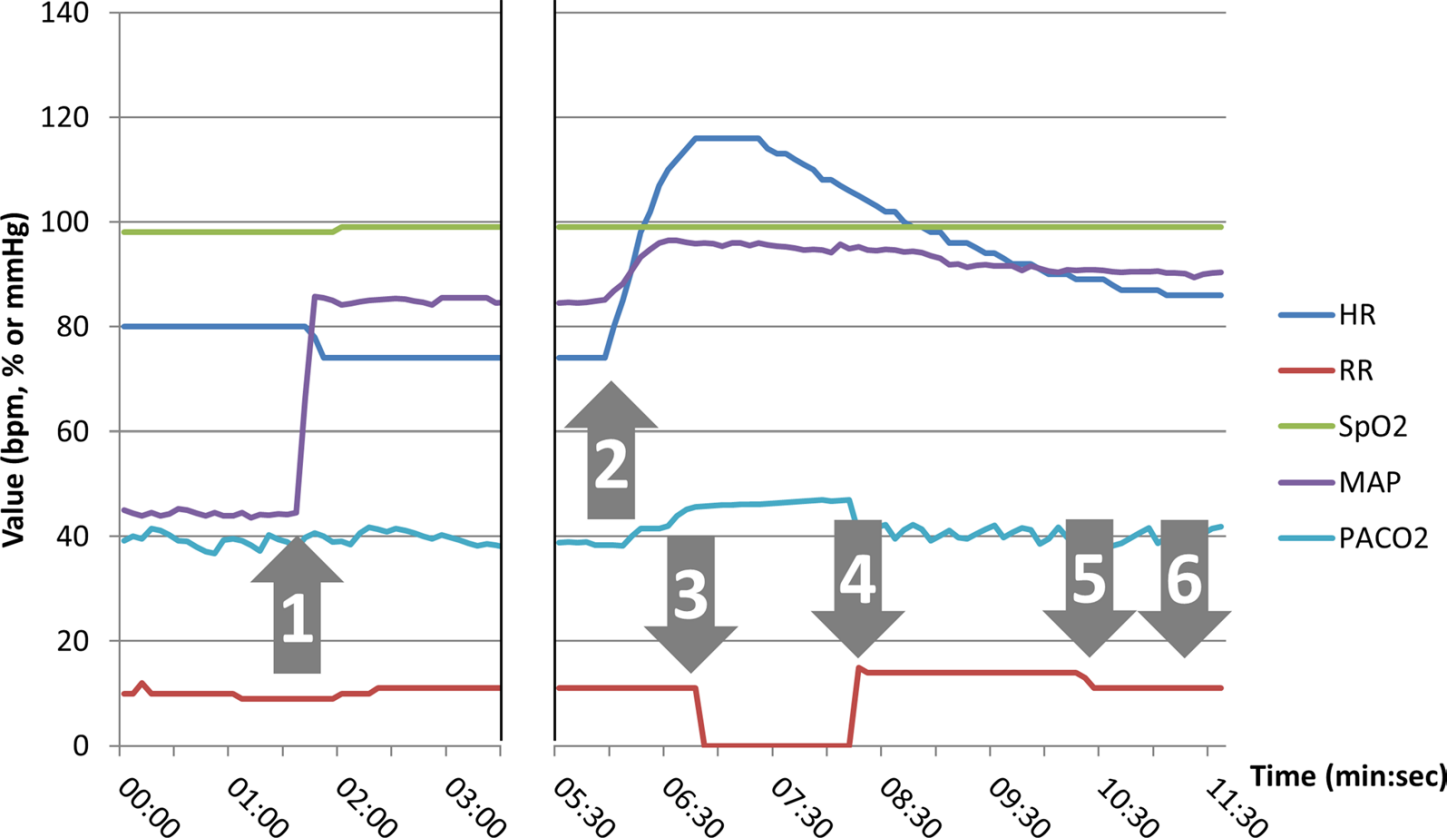
Example of the evolution of physiological parameters during one simulation. Annotated events: *#1* administration of 1000 mL of normal saline, *#2* induction of general anaesthesia, *#3* apnea, *#4* initiation of mechanical ventilation, *#5* reversion of muscle blockade, *#6* extubation. *HR* heart rate, *RR* respiratory rate, *SpO2* pulse oximetry, *MAP* mean arterial pressure, *PACO2* partial alveolar pressure of CO_2_

Figure 4 shows a typical example of the evolution of the physiological parameters during one of the sessions.

During the five simulation sessions, no severe complication of anaesthesia occurred as emulated by the simulator software: no cardiac arrest ensued, the lowest SpO2 recorded was 97 %, the lowest mean arterial pressure recorded (after initial restoration of hemodynamics) was 81.6 mmHg, no participant failed to intubate the mannequin within the imparted limits (5 min or 5 attempts).

**Fig. 5:**
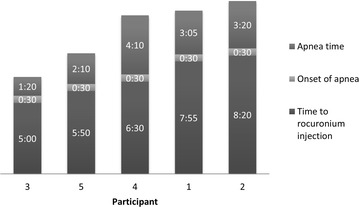
Time (minutes: seconds) between the beginning of the procedure and the muscle blocker injection, the onset of apnea and the apnea time, for all five participants

The median time to “incision” was 11 min 10 s, with a range from 6 min 50 s to 11 min 30 s (Fig. 5). The median apnea time was 3 min and 5 s (ranging from 1 min 20 s to 4 min 10 s). Three participants successfully intubated the mannequin at the first attempt, while the two others required a second attempt.

The objective skills assessment confirmed that most participants could appropriately conduct the critical tasks (Table 7).

**Table 7:** Skills assessment (N = 5)

Skill category	Median mark (±IQR)
Technical skills	16/20 (±1)
Non-technical skills	10/20 (±3)

The results of feedback received by the participants are summarized in Table 8.

**Table 8:** Feedback from the participants on the simulation material (−2: “bad”, −1:“mediocre”, 0:“average”, 1:“good”; 2: “excellent”)

	−2	−1	0	+1	+2
Quality of the briefing			1	3	1
Relevance of the scenario			1	3	1
Quality of the simulation equipment			1	2	2
Relevance of the research for space exploration				2	3
Was the software tool understandable?			2	2	1
Was the software tool self-sufficient?			3	2	

## Discussion

The experimental setting of this study was able to replicate, in a relevant environment, many of what are considered to be major constraints to anaesthesia delivery during a space exploration mission. This is, to the best of our knowledge, the first attempt to simulate complex medical procedures for spaceflight using the combination of a physical mannequin with advanced physiological modeling.

Our results suggest that crew members with very limited medical training were able to appropriately perform the first critical steps a basic general anaesthesia, meaning that after the initial management and according to the software, the simulated patient was hemodynamically stable, anaesthetised, intubated, ventilated and would have been able to undertake a surgical procedure. The protocols were carried out safely as no severe complication occurred. The objective skills assessment confirmed that the participants were able to conduct most technical tasks appropriately. Their performance on non-technical skills, which are often more difficult to acquire, was understandably poorer [23]. The feedback received from the participants confirmed that the scenario and simulation were engaging and of high quality and that the clarity of the software tool was good to excellent. The simplified anaesthetic procedure assessed in this study would be suitable for a very vast range of surgical scenarios, involving virtually any part of the body. Simpler and safer protocols such as conscious sedation could be proposed for superficial surgery or painful procedures (e.g. relocation of a dislocated limb).

Stating that conducting invasive medical procedures such as a rapid sequence induction and an intubation is safe without extensive medical training is a bold assumption. While several professional medical bodies do acknowledge that anaesthetic procedures can be carried out by non-anaesthetists or non-physicians, they insist that these providers must complete the appropriate training [2, 4, 6]. The situation is very different in many low-income countries throughout the world, where the shortage of medical professionals is such that anaesthetic and surgical procedures are regularly carried out by non-physician healthcare providers trained on the job, working alone and without the standard equipment and level of safety that is expected elsewhere [1, 3, 32]. In those conditions, a peri-operative mortality rate “only” two to three times higher than in high-resource settings can be seen as an achievement, which could be explained by at least two factors: the safety of ketamine-based anaesthesia (available in about three quarters of the hospitals) and the remarkable skills that the providers have managed to acquire in a very limited time [1, 3]. It makes little doubt that astronauts would possess the right skillset to carry such procedures, given that they receive the appropriate training during the preparatory phase of the mission.

Nevertheless, no analogue is capable of completely reproducing the unique conditions and challenges of a space exploration mission and this pilot study is indeed limited by a number of factors.

Several aspects of the study design could be discussed. First, the sample size, with just five participants, was particularly small, but this study represents a proof-of-concept experiment performed by a single crew at MDRS. The examined procedure is an oversimplification of the reality as it makes no doubt that the anaesthetic management of a bleeding unstable patient does not confine to the induction and the placement of an endotracheal tube. Several important tasks were simplified or bypassed, such as the haemodynamic management, the administration of blood products, the maintenance of anaesthesia for a prolonged period of time, surgery itself or post-operative care. Again, this was a pilot study focusing on intravenous induction and airway management. Of note, alterations in baroreflex and adrenergic receptors sensibility may complicate even further the management of an unstable astronaut [18, 33]. Agnew recommends to limit the use of adrenergic antagonists and possibly to increase the dose of alpha-adrenergic agonists [18].

The chosen scenario of an emergency laparotomy may appear far-streched. The ability to perform a laparotomy in space has however been identified as a core medical skill for low Earth orbit [34], and abdominal trauma represents a condition of high concern for interplanetary space missions [8]. While basic surgical skills can be acquired promptly [32], an emergency laparotomy performed by a non-physician in space cannot be considered straightforward. Damage control laparotomy in the context of a space mission has however been described by as “technically simple” and potentially “appropriate for telementoring”, which could include the use of pre-recorded video material [12]. It has anecdotally been performed by non-physicians [12]. We suggest that the surgical training for non-medical crew members could follow the same model as these anaesthetic techniques, combining reduced hands-on familiarization pre-flight with simplified procedures and smart tools for real-time guidance.

In a real life situation, the whole crew would be involved in the management of the injured individual. Our study design did not include any role for the other four team members, which again could be seen as a poor representation of the reality and did not allow to assess team-working skills. More advanced studies integrating this protocol into complete surgical procedures with more complex scenarios involving several simultaneous participants are planned. The short delay between the familiarization session and the actual simulation (4–6 days) could be criticized, because it could have helped with recalling. Because the crew rotations at the MDRS are only 2 weeks long, it was not technically feasible to extend this delay.

The validity and applicability of some of the results can be questioned. During the sessions, no desaturation occurred, but the reader must be aware that preliminary checks of the software revealed particularly slow desaturation times. With the appropriate preoxygenation, blood saturation reached 90 % after 8 min 55 s. Nevertheless, the longest apnea time in our study was 4 min 10 s, which is well below the physiological reserve [35]. The median time to incision of just above 11 min is most likely a poor estimate of how long it would take in an actual situation, because all the preparatory phases were skipped in our study. This task would require a significant amount of time for someone with no medical training, while carrying a substantial risk of error, for example in the drug dilution.

Finally, the main limitation of this study lies in the environment and the stress experienced by the participants. The breadth of the challenges posed by a situation like this happening during an actual mission is impossible to reproduce. Besides their multiple skills, astronauts are also selected for their ability to handle extreme stress, and it is undeniable that they would be among the best candidates, besides actual physicians, to be able to perform such advanced and invasive medical procedures in the most remote setting ever explored by humankind [12].

## Conclusions

The study design was able to reproduce many of the expected constraints to medical care delivery during a space exploration mission. The results suggest that non-medical personnel with minimal training may be able to perform the first steps of a basic anaesthetic procedure in a safe and efficient manner in an isolated environment with minimal equipment. The anaesthetic management of a bleeding and unstable patient encompasses many other factors that were simplified or bypassed in this preliminary pilot study, which nevertheless strengthens the validity of the simplified anaesthesia procedures developed specifically for space exploration missions. Personnel with limited medical training may be able to perform safely invasive procedures, which is in line with the current practice in many austere environments throughout the world. Astronauts are undeniably among the best candidates to be able to achieve such a challenging task. Further studies integrating this protocol into a comprehensive scenario are planned and will provide valuable input in designing medical systems for future space exploration missions.

## Abbreviations

ANTS: Anaesthetists’ Non-Technical Skills
HSO: Health and Safety Officer
MDRS: Mars Desert Research Station
